# Comparison of Esmolol and Dexmedetomidine Infusion in Attenuating Haemodynamic and Blood Glucose Response to Laryngoscopy and Intubation: A Single Blinded Study

**DOI:** 10.21315/mjms2021.28.3.4

**Published:** 2021-06-30

**Authors:** Vincent Koh, Saedah Ali, Mohamad Hasyizan Hassan, Ariffin Marzuki Mokhtar, Mohd Najib Majdi Yaacob, Mohd Zulfakar Mazlan

**Affiliations:** 1 Department of Anaesthesiology and Intensive Care, School of Medical Sciences, Universiti Sains Malaysia, Kubang Kerian, Kelantan, Malaysia; 2 Department of Anaesthesiology and Intensive Care, Hospital Universiti Sains Malaysia, Kelantan, Malaysia; 3 Biostatistic and Research Methodology Unit, School of Medical Sciences, Universiti Sains Malaysia, Kubang Kerian, Kelantan, Malaysia

**Keywords:** dexmedetomidine, laryngoscopy, intubation, haemodynamic response neuroendocrine stress

## Abstract

**Background:**

This study aims to compare the effect of infusions of two agents, dexmedetomidine and esmolol, with the control group in attenuating the haemodynamic stress response and neuroendocrine modulation surrogated by capillary blood glucose (BG) during the procedures.

**Methods:**

Sixty patients aged 18–70 years old who underwent elective surgeries involving endotracheal intubation were randomised into three groups of equal size: i) control; ii) dexmedetomidine and iii) esmolol. Heart rate (HR) was measured at baseline (T0), after drug administration (T1), after induction of anaesthesia (T2), immediately after intubation (T3), and 3 min, 5 min and 10 min after intubation (T4, T5 and T6). BG was measured pre-operatively and 30 min post-intubation.

**Results:**

Two-way repeated-measures analysis of variance showed significant time [within-group changes, *F*(3.2, 182.5) = 30.39, *P* < 0.001], treatment [between-group differences regardless of time, *F*(2, 57) = 50.24, *P* < 0.001] and interaction [between-group differences based on time, *F*(6.4, 182.5) = 37.65, *P* < 0.001] effects on HR. A significantly higher HR was observed in the control group compared to the dexmedetomidine and esmolol groups from T2 to T6. BG exhibited a significant time effect [*F*(1, 57) = 41.97, *P* < 0.001] with no significant treatment and interaction effects. All three groups showed a significant increase in BG from baseline.

**Conclusion:**

Both dexmedetomidine and esmolol are equally effective in attenuating haemodynamic responses to laryngoscopy and intubation. However, both do not significantly modulate neuroendocrine stress.

## Introduction

Laryngoscopy and tracheal intubation can lead to a profound sympathetic response, which has few consequences in healthy subjects but may result in unpredictable adverse effects such as arrhythmias, myocardial ischaemia, left ventricular failure, increased intracranial pressure or ruptured cerebral aneurysms in susceptible populations. Lately, the concern has not been emphasised on sympathetic responses alone but also potential neuroendocrine stress responses, which are thought to be associated with poor outcomes.

Attempts to attenuate the haemodynamic response to laryngoscopy and intubation using various pharmacological agents from different drug classes have been made. Yet none of the approaches are perfect and each has its limitations (1). Fentanyl, the most commonly used opioid for this purpose, is frequently given in insufficient doses, as higher doses are feared for their well-known complications (2). This has led to the exploration of the use of esmolol, an ultra-short-acting beta-1 blocker and dexmedetomidine, an alpha-2 agonist, to achieve optimal attenuation of these harmful sympathetic responses. The published studies in the literature have shown favourable outcomes for esmolol and dexmedetomidine with various administration doses and methods. However, few studies have been done to compare these two agents when administered periprocedurally as infusions.

Fentanyl is the usual co-induction agent for attenuating the haemodynamic stress response during laryngoscopy and tracheal intubation. However, the standard dosing used was found to be invariably insufficient. Arora et al. (2) concluded that the usual 2.0 μg/kg dose of fentanyl was unable to reduce the haemodynamic response to laryngoscopy and intubation, as the increase in systolic blood pressure (SBP) was 13.23%, diastolic blood pressure (DBP) was 9.42%, mean arterial pressure (MAP) was 12.78% and heart rate (HR) was 11.62% from baseline. Various agents from different classes have been studied extensively, including esmolol and dexmedetomidine, with most of these studies conducted using bolus administration instead of infusion. The latter is deemed to confer more stability and better control than a rapid bolus.

Liu et al. (3) studied the use of esmolol to control increases in HR and blood pressure during tracheal intubation after thiopentone and succinylcholine and concluded that esmolol infusion was effective in limiting elevations in HR, SBP and the rate pressure product, but did not entirely eliminate cardiovascular responses to intubation. A study done by Uysal et al. (4) in a hypertensive population found that dexmedetomidine administration before anaesthesia induction blunted the haemodynamic response to tracheal intubation and reduced the thiopental dose required; their results showed significantly lower percentage variation in HR, SBP and DBP than the esmolol and sufentanyl bolus groups. A similar study by Reddy et al. (5) assessing dexmedetomidine versus esmolol to attenuate the haemodynamic response to laryngoscopy and tracheal intubation showed that 1.0 μg/kg dexmedetomidine infusion before induction suppressed the haemodynamic response to a greater degree than that of a 2.0 mg/kg infusion of esmolol over 10 min.

Efe et al. (6) compared bolus and continuous infusion administration of esmolol on the haemodynamic response to laryngoscopy, endotracheal intubation and sternotomy in coronary artery bypass grafts and highlighted that esmolol infusion was more effective than esmolol bolus administration in controlling SBP during endotracheal intubation and sternotomy. A recent study by Jain et al. (7) compared the ability of dexmedetomidine and fentanyl to attenuate the haemodynamic response to laryngoscopy and tracheal intubation; they demonstrated that 1.0 μg/kg of dexmedetomidine infused over 10 min prior to the procedures was superior to 2.0 μg/kg fentanyl.

To date and to the best of our knowledge, there have not been any studies assessing neuroendocrine stress response modulation following laryngoscopy and intubation. A recent study using blood glucose (BG) estimation as an indirect surrogate of neuroendocrine stress has been conducted and assessed its modulation by dexmedetomidine or fentanyl during laparoscopic surgery (8), revealing a positive result. However, the study was mainly designed to assess responses to surgical stress or stimuli. Therefore, the current study’s primary aim was to compare the effects of esmolol and dexmedetomidine infusions in attenuating the sympathetic stress response and modulating the neuroendocrine stress response to laryngoscopy and tracheal intubation.

## Methods

### Study Design and Setting

This study was conducted as a single-centre, single-blinded and randomised controlled trial at the Universiti Sains Malaysia Hospital (HUSM). The hospital is a tertiary healthcare centre receiving referrals from government and private healthcare facilities in the northeast region of Peninsular Malaysia. After obtaining approval from the Human Research Ethics Committee of USM, the study was conducted from June 2018 to June 2019.

### Participants

Sixty patients aged 18–70 years old with American Society of Anesthesiologists (ASA) physical statuses I and II, who were planned to undergo various types of elective surgical procedures requiring endotracheal intubation, were recruited as study participants. Exclusion criteria included a body mass index of more than 30 kg/m^2^, pregnancy, cardiac or neurological insufficiency, anticipated difficult airway, metabolic syndrome, poorly controlled hypertension and patients on hormonal drugs. Written informed consent was obtained from all participants before their inclusion in the study.

### Randomisation and Blinding

The participants were divided into three equal-sized groups, namely the control, dexmedetomidine and esmolol groups. The randomisation sequence was prepared by a statistician not involved in the trial using the ‘psych*’* and ‘randomiser*’* packages of the R software, version 3.6.2 (R Project for Statistical Computing). Ten iterations of three groups with a block size of six were used to generate a random sequence using block randomisation. The random sequence was then concealed using sealed opaque envelopes labelled from 1–60. For each study participant, an envelope corresponding to the study participant’s identification number was opened by the investigator prior to to the insertion of the infusion pump. The participants were blinded to the procedure to which they were assigned.

### Anaesthesia Technique

Participants were premedicated orally with 3.75 mg/day midazolam before the operation. In the operating theatre, all participants were put on standard monitoring, which included measurements of non-invasive blood pressure, pulse oximetry, electrocardiography and capnography. Baseline values (the pre-anaesthetic reading) for HR, SBP, DBP, MAP and glucose level were recorded.

In the control group, no study drug was given. Participants in the dexmedetomidine group received a dexmedetomidine loading dose of 1.0 μg/kg over 10 min and maintenance at 0.4 μg/kg/h until 10 min post-intubation. Participants in the esmolol group received an esmolol infusion dose of 50 μg/kg/min, 10 min before the endotracheal intubation until 10 min post-intubation.

In the operating theatre, participants were pre-oxygenated for 3 min. An intravenous (IV) fentanyl (1.0 μg/kg–1.5 μg/kg) was given prior to induction of anaesthesia followed by IV propofol (1.0 mg/kg–2.0 mg/kg) dose until there was a loss of eye-lash reflex. Subsequently, IV rocuronium (0.9 mg/kg) was given to induce muscle relaxation approximately 60 sec before endotracheal intubation. Laryngoscopy was performed and followed by endotracheal intubation and general anaesthesia was maintained with 2% sevoflurane. At the end of the surgery, sevoflurane was discontinued and 100% oxygen was administered. The effects of the muscle relaxant were reversed using ivneostigmine (50 μg/kg) and glycopyrrolate (10 μg/kg).

### Outcome Assessment

Seven measurements of HR were recorded digitally on the operation theatre monitor: at baseline (T0), 1 min after administration of the study drug (T1), 1 min after induction of anaesthesia (T2), immediately after intubation (T3), 3 min after intubation (T4), 5 min after intubation (T5) and 10 min after intubation (T6). BG from blood samples was analysed using a glucometer pre-operatively (baseline) and at 30 min post-intubation.

### Statistical Analysis

IBM SPSS version 26.0 (IBM Corp., New York, United States of America) was used for all statistical analyses and the statistical significance was set at *P* < 0.05 (two-tailed). The statistical analyses of this study were performed on an intention-to-treat basis. Continuous variables were summarised as mean and standard deviation (SD), and categorical variables were summarised as frequency (*n*) and column percentage (%). Comparisons of socio-demographic data and baseline characteristics for all three groups were made using one-way analysis of variance (ANOVA) for continuous variables and the χ^2^ test for categorical variables.

Two-way repeated-measures ANOVA was conducted to determine the effect of the different interventions on HR and BG. Time and time-treatment interaction effects were assessed to determine within-group changes and between-group differences with regard to time. The Mauchly test of sphericity was used to check the sphericity assumption and a Greenhouse-Geisser epsilon correction was applied when the assumption was violated. Normality of the data was evaluated by assessment of studentised residuals using the Shapiro-Wilk test.

For both HR and BG, within-group changes in all three groups were determined separately using one-way repeated measures ANOVA following a significant overall time effect. Post-hoc analysis with a Bonferroni adjustment was then conducted to identify significant changes over time in each group. Statistically, significant time-treatment interaction effects indicated a significant difference in HR or BG over time among the three groups. Pairwise comparisons at each time point with Bonferroni adjustment were conducted to determine whether there were any differences in HR or BG between groups at each time point.

## Results

All 60 participants completed the study (20 participants in each group) with no adverse events reported. Comparisons of socio-demographic data and baseline characteristics for all participants in the three groups indicated that there were no significant differences (Table 1). The mean HR and BG levels throughout the measurement time are summarised in Table 2.

A two-way repeated-measures ANOVA was conducted to determine the effect of the different interventions on HR. Assessment of the studentised residuals using the Shapiro-Wilk test showed normality and no outliers (no studentised residuals exceeding ± three standard deviations). Mauchly’s test of sphericity [χ^2^(20) = 149.34, *P* < 0.001], indicated that the assumption of sphericity was violated, and therefore, a Greenhouse-Geisser epsilon correction was applied (ɛ = 0.534).

The analysis indicated that there was a significant overall time effect [*F*(3.2, 182.5) = 30.39, *P* < 0.001]. One-way repeated-measures ANOVA was conducted separately for all three groups to determine whether there were statistically significant changes in the mean HR from baseline (T0) to T6 within all the groups. Significant time effects were observed within all three groups [*F*(2.8, 53.6) = 33.09, *P* < 0.001 for the control group; *F*(3.2, 60.3) = 37.84, *P* < 0.001 for the dexmedetomidine group and *F*(2, 38.1) = 33.33, *P* < 0.001 for the esmolol group].

In the control group, the mean HR increased from 79.60 bpm at baseline (T0) to 80.85, 83.40, 94.35 and 95.75 bpm at T1 to T4, respectively. The mean HR then decreased to 90.50 and 86.55 bpm at T5 and T6, respectively. Post-hoc analysis with a Bonferroni adjustment revealed that the increase in mean HR from baseline (T0) was statistically significant from T3 to T6 (mean difference [MD] = 14.75; 95% CI: 8.23, 21.27; *P* < 0.001 at T3, MD = 16.15; 95% CI: 9.59, 22.71; *P* < 0.001 at T4, MD = 10.90; 95% CI: 4.64, 17.17; *P* < 0.001 at T5 and MD = 6.95; 95% CI: 0.51, 13.39; *P* = 0.026 at T6).

On the other hand, in the dexmedetomidine group, the mean HR exhibited a decreasing trend from 79.40 bpm at baseline (T0) to 77.95, 73.05, 69.60, 64.20, 59.90 and 59.40 bpm at T1 to T6, respectively. Post-hoc analysis with a Bonferroni adjustment revealed that the reduction in the mean HR from baseline (T0) was statistically significant from T2 to T6 (MD = 6.35; 95% CI: 0.28, 12.42; *P* = 0.035 at T2, MD = 9.80; 95% CI: 2.03, 17.56; *P* = 0.006 at T3, MD = 15.20; 95% CI: 10.01, 20.39; *P* < 0.001 at T4, MD=19.50; 95% CI: 12.89, 26.11; *P* < 0.001 at T5 and MD = 20.00; 95% CI: 12.31, 27.69; *P* < 0.001 at T6).

In the esmolol group, the mean HR slightly increased from 77.45 bpm at baseline (T0) to 77.90 bpm at T1, before it decreased to 71.45, 71.40, 68.30, 64.75 and 62.50 bpm at T2 to T6, respectively. Like the dexmedetomidine group, post-hoc analysis with a Bonferroni adjustment revealed that the reduction in mean HR from baseline (T0) within the esmolol group was statistically significant from T2 to T6 (MD = 6.00; 95% CI: 1.01, 10.99; *P* = 0.010 at T2, MD=6.05; 95% CI: 0.13, 11.97; *P* = 0.042 at T3, MD = 9.15; 95% CI: 2.57, 15.73; *P* = 0.002 at T4, MD = 12.70; 95% CI: 5.79, 19.61; *P* < 0.001 at T5, and MD = 14.95; 95% CI: 7.08, 22.82; *P* < 0.001 at T6).

The test of between-subject effects indicated that there was a significant difference in HR between the three groups regardless of time (treatment effect) [*F*(2, 57) = 50.24, *P* = < 0.001]. Pairwise comparisons revealed that the overall HR was significantly higher in the control group compared to the dexmedetomidine (MD = 18.21; 95% CI: 13.23, 23.20; *P* < 0.001) and esmolol groups (MD = 16.75; 95% CI: 11.77, 21.73; *P* < 0.001). No significant difference in HR was observed between the dexmedetomidine and esmolol groups (MD = −1.46; 95% CI: −6.45, 3.52; *P* > 0.95).

There was a statistically significant interaction effect between the intervention group and time on mean HR [*F*(6.4, 182.5) = 37.65, *P* < 0.001]. Pairwise comparisons at each time point with Bonferroni adjustments revealed that the HR of participants in the control group was significantly higher than those of participants in the dexmedetomidine group from T2 to T6 (MD = 10.35; 95% CI: 4.38, 16.33; *P* < 0.001 at T2, MD = 24.75; 95% CI: 18.58, 30.92; *P* < 0.001 at T3, MD = 31.55; 95% CI: 25.63, 37.48; *P* < 0.001 at T4, MD = 30.6; 95% CI: 25.21, 35.99; *P* < 0.001 at T5, and MD = 27.15; 95% CI: 21.25, 33.05; *P* < 0.001 at T6).

**Figure 1:**
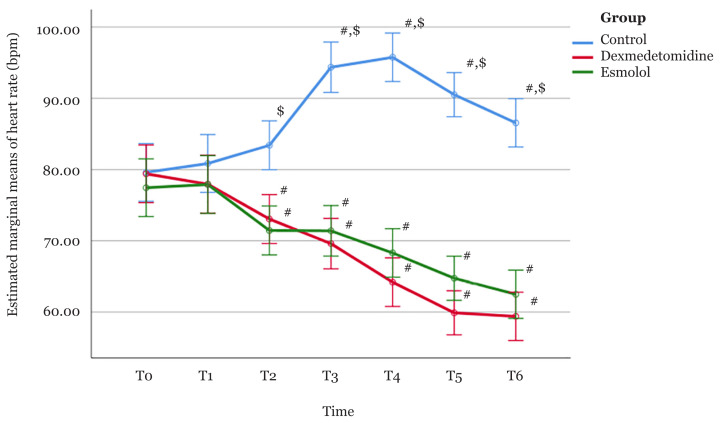
Profile plot showing estimated marginal means of heart rate for the control, dexmedetomidine and esmolol groups. The error bars represent the 95% confidence intervals of the means Notes: # = a significant within-group change from T0; $ = a significant between-group difference

A similar pattern of differences was observed between the control and esmolol groups (MD = 11.95; 95% CI: 5.98, 17.93; *P* < 0.001 at T2, MD = 22.95; 95% CI: 16.78, 29.12; *P* < 0.001 at T3, MD = 27.45; 95% CI: 21.53, 33.38; *P* < 0.001 at T4, MD = 25.75; 95% CI: 20.36, 31.14; *P* < 0.001 at T5, and MD = 24.05; 95% CI: 18.15, 29.95; *P* < 0.001 at T6). There was no significant difference in HR between the dexmedetomidine and esmolol groups throughout the entire period of measurement. The within-group changes and between-group differences in HR between the three intervention groups are illustrated in Figure 1.

A two-way repeated-measures ANOVA was conducted to determine the effect of the different interventions on BG. Assessment of the studentised residuals using the Shapiro-Wilk test showed normality and no outliers (no studentised residuals exceeding ± three standard deviations). Mauchly’s test of sphericity was not applicable since BG was measured only twice.

The analysis indicated that there was a significant overall time effect [*F*(1, 57) = 41.97, *P* < 0.001]. A one-way repeated-measures ANOVA was conducted for each group to determine whether there were any significant changes in BG within the three groups. Significant time effects were observed within all three groups [*F*(1, 19) = 22.65, *P* < 0.001 for the control group, *F*(1, 19) = 11.66, *P* = 0.002 for the dexmedetomidine group and *F*(1, 19) = 9.96, *P* = 0.005 for the esmolol group].

In the control group, the mean BG increased significantly from 5.89 mg/dL to 6.91 mg/dL (MD = 1.02; 95% CI: 0.57, 1.46; *P* < 0.001). A similar pattern was observed in the other two groups. The mean BG increased from 5.93 mg/dL to 7.01 mg/dL (MD = 1.08; 95% CI: 0.45, 1.71; *P* < 0.001) in the dexmedetomidine group and increased from 5.98 mg/dL to 6.56 mg/dL in the esmolol group (MD = 0.58; 95% CI: 0.20, 0.97; *P* = 0.005).

**Figure 2:**
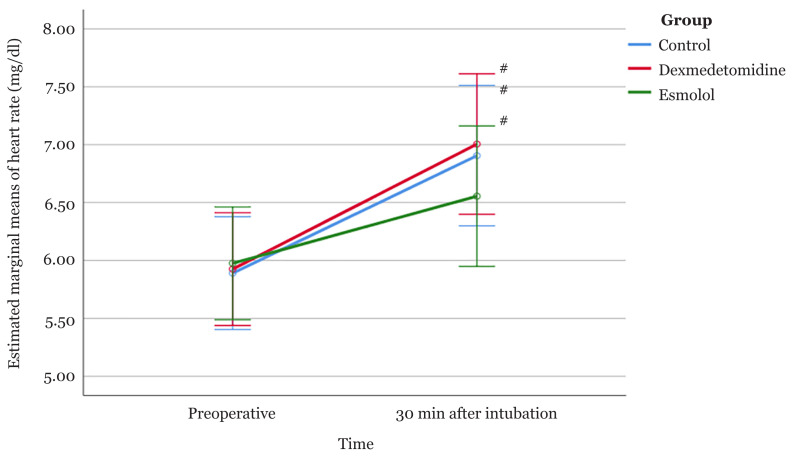
Profile plot showing estimated marginal means of blood glucose for participants in the control, dexmedetomidine and esmolol groups. The error bars represent the 95% confidence intervals of the means Note: # = a significant within-group change from the pre-operative measurement

The tests of between-subject effects indicated that there were no significant differences in BG among the three groups regardless of time (treatment effect) [*F*(2, 57) = 0.17 and *P* = 0.845]. Similarly, the interaction between the intervention group and time on mean BG was not statistically significant [*F*(2, 57) = 1.30, *P* = 0.739], indicating no significant difference in BG level between the three groups pre-operatively and 30 min after intubation (Figure 2).

## Discussion

The haemodynamic changes resulting from laryngoscopy and endotracheal intubation following induction of anaesthesia have been well documented. They are thought to be secondary to reflex sympathetic discharge caused by laryngopharyngeal stimulation, which commonly manifests as hypertension and tachycardia mediated by cardiac accelerator nerves and sympathetic chain ganglia (9). Reid and Brace (10) first described these haemodynamic changes brought about by laryngoscopy and intubation. These changes are usually transient and well tolerated by normal patients, but not necessarily in a susceptible population such as cardiovascular or cerebrovascular insufficiency patients. Thus, various drug regimens and techniques, including beta-blockers and alpha-2 agonists, have been studied at different doses and with different administration methods to compare their efficacies in attenuating these haemodynamic responses following laryngoscopy and endotracheal intubation. Concerning modulation of neuroendocrine stress responses using BG estimation as an indirect assessment, only a single trial by Gupta et al. (8) showed that dexmedetomidine was efficacious compared to fentanyl premedication during laparoscopic cholecystectomy under general anaesthesia.

In this study, there was a significant increase in mean HR at laryngoscopy in the control group. In both the dexmedetomidine and esmolol groups, a significant reduction in HR was observed from 1 min after anaesthesia until 10 min after intubation when compared to the control group. This result is consistent with the studies done by Sharma et al. (11), Jain et al. (7) and Karuppiah et al. (1). In the control group, HR increased from immediately after post intubation to 3 min post intubation and was comparable with the basal value at 5 min and 10 min post-intubation. In the dexmedetomidine and esmolol groups, a significant decrease in HR was observed at 1 min after anaesthesia to 10 min post-intubation compared to the control group. These findings are similar to those of Sharma et al. (11), Jain et al. (7) and Karuppiah et al. (1).

At almost all time points, dexmedetomidine and esmolol were found to elicit consistently significant differences in all haemodynamic parameters from immediately after post intubation until 10 min post-intubation. These findings are similar to Reddy et al. (5) and Sharma et al. (11). Both studies concluded that dexmedetomidine is more effective than esmolol in attenuating haemodynamic responses or maintaining haemodynamic stability following laryngoscopy and intubation.

Regarding BG pre-operatively and at 30 min post-intubation, all three groups showed increases in the post-intubation period. However, the increase in BG was slightly less pronounced in the esmolol group than in the control and dexmedetomidine groups. Nonetheless, neither drug was able to completely prevent increases in BG up to 10 min measurement.

In Liu et al.’s study (3), the authors used an esmolol infusion of 500 μg/kg/min for 4 min and then 300 μg/kg/min for 8 min, whereas Ghaus et al. (12) used an esmolol infusion of 300 μg/kg/min for 4 min followed by 200 μg/kg/min for 6 min. Both studies concluded that esmolol attenuates the haemodynamic response to laryngoscopy and intubation. Our study used a much lower infusion dose of 50 μg/kg/min starting from 10 min before laryngoscopy and intubation, until 10 min post-intubation. Our findings were shown to be consistent with the aforementioned two studies.

As for dexmedetomidine, Sebastian et al. (13) demonstrated that an intravenous dose of 0.75 μg/kg is the optimal dose to attenuate the stress response to laryngoscopy and endotracheal intubation. Modh et al. (14) also observed that 1.0 μg/kg dexmedetomidine infusion over 10 min provides effective and complete attenuation of pressor responses to laryngoscopy and intubation. Our study demonstrated a consistent, comparable result with a dexmedetomidine loading dose of 1.0 μg/kg over 10 min, which was maintained at 0.4 μg/kg/hour until 10 min post-intubation.

Our study has a few limitations. This is a single-centre study; however, it can serve as a template protocol for the continuation or extension of similar clinical trials in the future. Additionally, plasma catecholamine level monitoring was not carried out due to limited facilities in our centre. Further larger studies are needed to evaluate the relationship between esmolol and its potential for neuroendocrine modulation.

## Conclusion

This study demonstrates that dexmedetomidine and esmolol are equally effective in attenuating haemodynamic responses (i.e. heart rate) to laryngoscopy and intubation. We conclude that a dose of esmolol infusion as low as 50 μg/kg/min is as effective as a dexmedetomidine infusion involving a 1.0 μg/kg loading dose followed by maintenance at 0.4 μg/kg/h. Both dexmedetomidine and esmolol did not significantly modulate the neuroendocrine stress response to laryngoscopy and intubation; however, esmolol resulted in a smaller increase in BG.

## Figures and Tables

**Table 1:** Socio-demographic data and baseline characteristics of study participants by group (*n* = 60)

Variables	Group			Test statistic (df)	*P*-value

	Control (*n* = 20)	Dexmedetomidine (*n* = 20)	Esmolol (*n* = 20)		
Age (years)	39.70 (14.78)	43.25 (18.26)	37.40 (14.17)	0.69 (2, 57)	0.504^a^
BMI (kg/m^2^)	24.99 (2.51)	25.75 (2.63)	24.37 (3.90)	0.99 (2, 56)	0.376^a^
Sex					
Male	12 (60.0)	12 (60.0)	10 (50.0)	0.54 (2)	0.762^b^
Female	8 (40.0)	8 (40.0)	10 (50.0)		
Race					
Malay	10 (50.0)	14 (70.0)	15 (75.0)	3.07 (2)	0.215^b^
Non-Malay	10 (50.0)	6 (30.0)	5 (25.0)		
ASA score					
I	10 (50.0)	9 (45.0)	11 (55.0)	0.40 (2)	0.819^b^
II	10 (50.0)	11 (55.0)	9 (45.0)		

Notes: BMI = body mass index, ASA = American Society of Anesthesiologists physical status.

a One-way ANOVA test *P*-value,

b χ^2^-test *P*-value.

The age and BMI variables were presented as mean and standard deviation (SD), whereas sex, race and ASA score were presented as frequency (*n*) and percentage (%)

**Table 2:** Mean heart rate and blood glucose levels at each time point (*n* = 60)

Variables	Group		

	Control (*n* = 20)	Dexmedetomidine (*n* = 20)	Esmolol (*n* = 20)
Heart rate (bpm)			
T0 (before drug administration)	79.60 (8.73)	79.40 (9.10)	77.45 (9.30)
T1 (after drug administration)	80.85 (8.16)	77.95 (10.52)	77.90 (8.30)
T2 (after induction of anaesthesia)	83.40 (7.60)	73.05 (8.92)	71.45 (6.22)
T3 (immediately after intubation)	94.35 (8.48)	69.60 (7.57)	71.40 (7.64)
T4 (3 min after intubation)	95.75 (8.52)	64.20 (6.40)	68.30 (7.72)
T5 (5 min after intubation)	90.50 (7.46)	59.90 (6.50)	64.75 (6.72)
T6 (10 min after intubation)	86.55 (7.76)	59.40 (6.970)	62.50 (7.94)
Blood glucose (mg/dL)			
Pre-operative	5.89 (1.10)	5.93 (1.06)	5.98 (1.10)
30 min post-intubation	6.91 (1.47)	7.01 (1.32)	6.56 (1.26)

Notes: Both variables were presented as mean and standard deviation (SD)
